# Vomiting Blood, Missing the Heart: A Rare Presentation of Acute Myocardial Infarction

**DOI:** 10.7759/cureus.95126

**Published:** 2025-10-22

**Authors:** Suad Albulushi, Nina Farazan, Yasmeen AlHarmali, Ahmadreza Bagheri

**Affiliations:** 1 Emergency Medicine, Al Nahdha Hospital, Muscat, OMN; 2 Clinical Emergency and Global Health Education, University of Toronto, Toronto, CAN; 3 Medical Education/Simulation, Administration and Medical Education, MGH Institute of Health Professionals, Boston, USA; 4 Emergency Medicine, Oman Medical Specialty Board, Muscat, OMN; 5 Emergency Medicine, Al Nahdha Hospital, Ministry of Health, Muscat, OMN; 6 Emergency Medicine, Medical City Hospital for Military and Security Services, Muscat, OMN; 7 Cardiac/Thoracic/Vascular Surgery, The Royal Hospital, Muscat, OMN

**Keywords:** acute coronary syndrome, chest pain, hematemesis, multi-disciplinary team, myocardial infarction, upper gastrointestinal bleeding

## Abstract

Hematemesis, the vomiting of blood, is an uncommon initial manifestation of myocardial infarction (MI). We describe a case involving the clinical progression of a 46-year-old man who arrived at the emergency department with symptoms including active coffee ground vomitus and severe epigastric pain. While acute coronary syndrome (ACS) typically manifests with chest pain, this case highlights the significance of considering MI even in the absence of this hallmark symptom. The patient's symptoms initially raised suspicion of upper gastrointestinal bleeding. However, given the patient's cardiovascular risk factors, acute MI (AMI) was considered, confirmed by abnormal ECG and elevated troponin levels. The patient received percutaneous coronary intervention (PCI) and was discharged with dual antiplatelet therapy. The case stresses the necessity of a comprehensive differential diagnosis when assessing patients with symptoms similar to gastrointestinal bleeding, as timely recognition of atypical presentations of MI is crucial for favorable outcomes. This report aims to raise awareness of the importance of comprehensive evaluation and tailored management strategies for atypical presentations of ACS. Further research is needed to guide optimal approaches in these challenging scenarios.

## Introduction

Acute coronary syndrome (ACS) includes a range of clinical presentations; some presentations may not prominently include chest pain. While chest pain is frequently reported as the primary symptom in cases of acute myocardial infarction (AMI), not all instances of ACS manifest with this characteristic complaint. The classical presentation of ischemia is often depicted as a squeezing sensation of substantial chest pressure, described as a "burning" discomfort, or accompanied by dyspnea. In a minority of cases, chest pain may exhibit atypical features. Atypical presentations are particularly common among older individuals, patients with diabetes, and women [1-3]. The occurrence of complications involving gastrointestinal bleeding and ACS is not infrequent.

In this case report, we outline the clinical course of a 46-year-old male who presented to the emergency department with active coffee ground vomitus and severe epigastric pain that began approximately three hours earlier. This case underscores the critical importance of considering a diagnosis of AMI even in the absence of chest pain. It highlights the complexities involved in evaluating patients with myocardial infarction (MI) who do not exhibit the typical symptoms.

## Case presentation

A 46-year-old Arabian male arrived at our emergency department in the evening, reporting hematemesis that commenced three hours prior, accompanied by abdominal pain and diarrhea. On the day of presentation, he experienced acute, severe epigastric pain, without concomitant shortness of breath or diaphoresis. Subsequently, he experienced three episodes of loose stools, during which he was uncertain about the color. Notably, he was observed holding a yellow bag containing coffee-ground vomitus. The patient presented with severe, persistent abdominal pain rated 9/10 on the numeric rating scale, accompanied by continuous vomiting and an inability to lie supine due to pain. He appeared distressed, with eyes tightly closed, but denied pain radiating beyond the abdominal region. There was no history of recent ingestion of raw or undercooked food. His past medical history included hypertension, diabetes mellitus, and asthma, with no history of smoking or alcohol use.

At the time of presentation, his vital signs were recorded as follows: Glasgow Coma Scale score of E4V5M6; temperature at 36.6°C; blood pressure at 115/110 mmHg; heart rate of 101 beats per minute; respiratory rate of 24 breaths per minute; SpO_2 _at 98% on room air; and a random blood sugar level of 25 mmol/dL or 450 mg/dL.

Furthermore, there was no evidence of cervical lymphadenopathy, nor was pharyngeal erythema observed. Pulmonary auscultation revealed normal findings. The abdomen was flat and soft, exhibiting hyperactive bowel sounds. Tenderness was detected on palpation of the upper abdomen. There were no signs of cold extremities or edema in the lower limbs.

**Figure 1:**
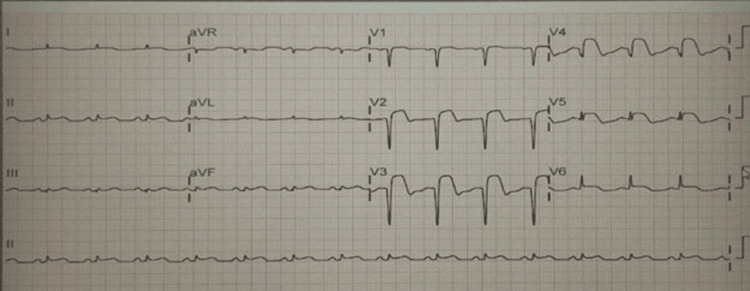
Electrocardiogram before intervention

The patient's symptoms upon arrival in the emergency room initially suggested the possibility of upper gastrointestinal bleeding. A gastroenterology consultation determined that the blood in the patient’s vomit is due to a Mallory-Weiss tear caused by high-pressure vomiting, and urgent endoscopy is not required. His initial chest X-ray was normal. Management began with the administration of 80 mg of omeprazole, followed by a continuous infusion at 8 mg/hour, in addition to the use of an antiemetic medication. Blood tests were promptly sent for further evaluation. AMI was included in the differential diagnosis due to the patient's cardiovascular risk factors, which included hypertension, diabetes, and being male. An immediate electrocardiogram (ECG) was deemed necessary. The ECG showed abnormal Q waves and ST elevation in leads V2-V5, as well as in leads II and III (Figure 1).

Blood tests revealed elevated troponin levels (1,440 pg/mL), while kidney function tests and haemoglobin were within normal limits (Table 1). The patient diagnosed with MI received an initial heparin bolus of 60 U/kg (maximum 4,000 U), followed by a continuous infusion of 12 U/kg/hour (maximum 1,000 U/hour) and Plavix 300 mg loading dose as prescribed by the cardiology consultant. The decision was made to transfer the patient, who remained vitally and clinically stable, to a hospital with catheterization laboratory capabilities within 120 minutes.

**Table 1:** Lab tests

Venous Blood Gas	Patient		Reference Range
pH	7.34		7.32-7.42
HCO_3_	24 mmol/L		22-28
PCo_2_	45 mmHg		40-50
PO_2_	37 mmHg		30-50
Other Tests	Patient		Reference Range
Troponin T	1440 ng/L		0-14
Bilirubin Total	22 umol/L		3-19
ALT	39 U/L	4-40	
ALP	107 U/L	36-104	
Protein Total in Serum	83 g/L	60-80	
Albumin in Serum	45 g/L	35-50	
Globulin Gap in Serum	38 g/L	24-35	
Amylase in Plasma	126 U/L	28-100	
C-Reactive Protein	10 mg/L	0-5	
Na^+^	137 mmol/L	135-145	
K^+^	4.6 mmol/L	3.5-5	
Cl^-^	93 mmol/L	95-110	
HCO_3_	21 mmol/L	22-28	
Creatinine	111 umol/L	62-106	
Urea	9.4 mmol/L	2.5-7.5	
Anion Gap	28 mmol/L	7-17	
eGFR.MDRD.	62 Ml/min/1.73m^2^		
WBC	9.35 *10^9 ^L	2.2-10	
RBC	6.26 *10^6^ uL	4.5-5.8	
Hb	15.10 g/dL	11.5-15.5	
Plt	184 *10^3^ uL	150-450	
MCV	75.3 fL	78-96	
MCH	24.1 pg	26-33	

Coronary angiography revealed the following findings: Right coronary artery (RCA): 30% stenosis in the proximal part and 80% stenosis at the mid part, with the distal segment being normal; left main coronary artery (LMCA): normal; left anterior descending artery (LAD): 50% stenosis in the proximal part and 90% stenosis with two thrombolysis in MI (TIMI) flow, with the diagonal branch being normal; and left circumflex artery (LCX): normal.

For a patient with MI and over 70% narrowing of the RCA, stress test echocardiography is contraindicated and can proceed directly with intervention through angioplasty. Percutaneous coronary intervention (PCI) was carried out on the LAD, involving the placement of two drug-eluting stents (DES) (see Videos 1-2).

**Video 1:** LAD before stent LAD: Left anterior descending artery

**Video 2:** LAD after stent LAD: Left anterior descending artery

**Figure 2:**
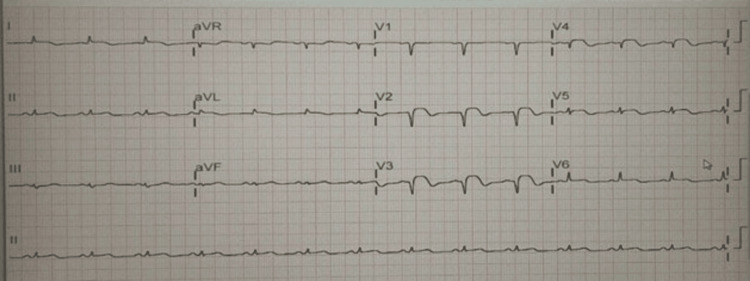
Electrocardiogram after intervention

After the procedure, the patient was admitted to the intensive care unit. A post-procedure electrocardiogram (ECG) indicated an improvement in ST elevation compared to the initial ECG (Figure 2).

Dual antiplatelet therapy was initiated, and the omeprazole infusion was continued. The coronary care unit (CCU) team consulted the surgical team to explore other potential causes of the abdominal pain. Hence, an abdominal computed tomography angiography scan was performed at this time and revealed no abnormal findings, except for diffuse wall thickening of the stomach with submucosal edema.

Following admission, the patient's abdominal pain persisted, although with a change in the character of vomiting from hematemesis to non-bloody emesis. The surgical team signed out, as the CT findings did not indicate any surgical emergency. The patient was then referred to the gastroenterology team for further evaluation. The gastroenterology team considered the following: The initial hematemesis may have been secondary to Mallory-Weiss syndrome due to repetitive vomiting. No urgent esophagogastroduodenoscopy (OGD) was warranted, given the stable haemoglobin (Hb) levels. The patient’s overall condition remained stable. A second percutaneous coronary intervention (PCI) was performed for the patient, this time targeting the RCA.

The DES was placed (see Video 3). Post-PCI, an echocardiogram was conducted and showed (see Videos 4-5): Normal LV in size with reduced ejection fraction (EF: 35%, moderate systolic dysfunction); akinetic thinned out mid-anterior septum; mid-apical IVS, apex, apical lateral, mid-apical inferior wall, and apical anterior wall; Grade I diastolic dysfunction; normal right ventricular size and systolic function; mildly calcified mitral valve with mitral regurgitation; no stenosis; and trivial tricuspid regurgitation. Organized thrombus is seen in the LV apex, measuring 19 × 11 mm.

**Video 3:** RCA before and after stent RCA: Right coronary artery

**Video 4:** Parasternal long-axis view ECHO post PCI PCI: percutaneous coronary intervention

**Video 5:** Four-chamber apical view ECHO post PCI PCI: percutaneous coronary intervention

The patient recovered without any complications or new issues and was discharged on the sixth day of hospitalization, prescribed acetylsalicylic acid (ASA) 80 mg daily, Plavix 75 mg daily, and rivaroxaban 20 mg daily for one week due to left ventricular clot formation. After one week, aspirin (or ASA) was discontinued, and the patient was maintained on rivaroxaban and clopidogrel (Plavix) for an additional three months. For heart failure with reduced ejection fraction (HFrEF), dopamine was prescribed for inotropic support to improve cardiac output, while sacubitril/valsartan was initiated to reduce hospitalization risk and enhance outcomes by increasing natriuretic peptide effects and promoting vasodilation.

## Discussion

Summary of diagnostic dilemma

An urgent ECG revealed diffuse ST-segment elevation MI (STEMI) involving nearly all leads. It was uncertain whether to administer antiplatelet therapy as in typical MI cases, given the unclear distinction between a type 1 and type 2 (demand-related) myocardial infarction in the context of active bleeding.

While chest pain is the typical symptom of AMI, atypical complaints or signs such as left shoulder pain, pharyngeal pain, earache, headache, back pain, syncope, and dyspnea can also be associated with AMI. When atypical symptoms or signs are present, there is a propensity for delays in diagnosis and treatment, potentially resulting in adverse outcomes [4,5].

Even with progress in cardiovascular disease treatment, differentiating between chest pain of cardiac origin and non-cardiac sources remains a diagnostic challenge. Studies indicate that 8.4%-33% of MI cases present without chest pain, with 24.3% of these patients experiencing vomiting [6,7]. Additionally, MI without chest pain often requires a longer time for diagnosis compared with typical presentations and is linked to a worse prognosis [7].

The management of concurrent hematemesis and AMI remains controversial because the therapeutic modalities for each condition may conflict. Administering antiplatelet therapy for MI may exacerbate bleeding, whereas prioritizing hemostasis may worsen myocardial ischemia. Previous reports have demonstrated that cardiac complications are common among patients admitted to intensive care with upper gastrointestinal bleeding (UGIB) [1] and that endoscopy can often be performed safely when closely coordinated with cardiology [2,3]. Nevertheless, balancing ischemic and hemorrhagic risk remains complex [8,9].

In this patient, the gastrointestinal manifestations - abdominal pain, bloody vomiting, and diarrhea - complicated the differentiation between MI and abdominal causes. In the emergency department, the initial suspicion frequently leans toward UGIB. However, when diagnostic uncertainty persists despite suggestive gastrointestinal symptoms, clinicians should maintain a high index of suspicion for MI, particularly in elderly or diabetic patients.

According to the 2023 European Society of Cardiology (ESC) and 2025 American College of Cardiology/American Heart Association (ACC/AHA) guidelines, decisions regarding dual antiplatelet therapy (DAPT) and anticoagulation should be individualized in the setting of active bleeding [8,9]. Both guidelines emphasize multidisciplinary coordination, especially between cardiology and gastroenterology teams, to balance ischemic and hemorrhagic risks. Life-threatening bleeding warrants temporary withholding or de-escalation of antithrombotic therapy until hemostasis is achieved, followed by early re-initiation once bleeding risk stabilizes [8,9].

This case underscores the importance of patient-centered decision-making, effective interdisciplinary communication, and adherence to guideline-directed individualized management to achieve both hemostatic control and myocardial protection.

## Conclusions

This case underscores the importance of maintaining a high index of suspicion for atypical presentations of ACS and adapting management strategies to address concurrent clinical challenges, such as UGIB. Both patient-related factors (e.g., advanced age, comorbidities) and procedural factors (e.g., medications administered during angioplasty) can increase the risk of UGIB in this population. Recognizing these risks - along with understanding their incidence, prognostic implications, and impact on outcomes - is essential for identifying high-risk patients and tailoring individualized treatment strategies. For emergency care providers, awareness of ACS presentations that deviate from the classic chest pain profile, particularly those involving gastrointestinal symptoms such as epigastric pain, vomiting, or hematemesis, is critical. These atypical manifestations are more prevalent among older adults, women, and patients with diabetes, and they frequently pose diagnostic challenges. Timely recognition is vital, as ACS without characteristic chest pain is associated with delayed diagnosis and poorer prognosis.

Furthermore, interdisciplinary collaboration is paramount when managing patients with concurrent ACS and UGIB. Effective coordination among cardiology, gastroenterology, and emergency medicine teams can optimize diagnostic accuracy, minimize complications, and improve patient outcomes. According to the 2023 ESC and 2025 ACC/AHA guidelines, management decisions regarding DAPT and anticoagulation should be individualized in the setting of active bleeding, with a focus on multidisciplinary coordination and early therapy re-initiation after hemostasis. Ultimately, this case highlights the need for continued research and clinical awareness to refine evidence-based strategies for managing complex presentations of ACS with concurrent UGIB. Applying these insights in clinical practice can enhance the comprehensiveness of patient assessment, reduce diagnostic delays, and improve outcomes for patients presenting with atypical and high-risk cardiovascular emergencies.
